# Retraction: Chicory (*Cichorium intybus* L.) Root Extract Regulates the Oxidative Status and Antioxidant Gene Transcripts in CCl_4_-Induced Hepatotoxicity

**DOI:** 10.1371/journal.pone.0173587

**Published:** 2017-03-06

**Authors:** Yasser S. El-Sayed, Mohamed A. Lebda, Mohammed Hassinin, Saad A. Noeman

The authors and editors retract this publication [1] due to concerns about the data presented in Figure 1.

After the publication of the article, we were notified of concerns about Figure 1. Specifically, it was brought to our attention that Figure 1 appears to be composed of multiple gel fragments spliced together in a single image. Furthermore, this figure was published previously in two articles in The Egyptian Journal of Biochemistry and Molecular Biology [2] [3], where the data were reported to represent different experiments. One of these articles was subsequently retracted due to these same concerns.

The authors were not able to provide the original, uncropped gel images used to generate this figure or to reproduce the results using the same experimental conditions. In light of these concerns, the authors and the *PLOS ONE* Editors retract this article.

Note that the last author’s name is misspelled on the published article. The correct spelling is Saad A. Noeman.

## References

[1] El-Sayed YS, Lebda MA, Hassinin M, Neoman SA (2015) Chicory (*Cichorium intybus* L.) Root Extract Regulates the Oxidative Status and Antioxidant Gene Transcripts in CCl_4_-Induced Hepatotoxicity. PLoS ONE 10(3): e0121549 doi: 10.1371/journal.pone.0121549 25807561 10.1371/journal.pone.0121549 PMC4373694

[2] Soliman NA, Noeman SA (2014) CCl4-INDUCED HEPATOTOXICITY: PROTECTIVE EFFECTS OF CARNOSINE ON PEROXISOME PROLIFERATOR-ACTIVATED RECEPTOR GAMMA GENE EXPRESSION, GENOTOXICITY AND OXIDANT/ANTI OXIDANT STATUS IN RATS. Egyptian Journal of Biochemistry & Molecular Biology 32(1): 115–131. [Retracted 2016, http://www.ajol.info/index.php/ejbmb/article/view/146920]

[3] Keshk WA, Noeman SA (2013) β-ALANYL-L-HISTIDINE, AN ANTI-OXIDANT, ANTIFIBROTIC AND GENO/CYTOTOXICITY INHIBITOR: A SHIELD AGAINST CCl4-INDUCED HEPATOTOXICITY IN RATS. Egyptian Journal of Biochemistry & Molecular Biology 32(1): 99–114

